# Clinical relevance of lipid panel and aminotransferases in the context of hepatic steatosis and fibrosis as measured by transient elastography (FibroScan®)

**DOI:** 10.5937/jomb0-24689

**Published:** 2021-01-26

**Authors:** Luis Alberto Chi-Cervera, Gordon Iaarah Montalvo, María Eugenia Icaza-Chávez, Julio Torres-Romero, Víctor Arana-Argáez, Mario Ramírez-Camacho, Julio Lara-Riegos

**Affiliations:** 1 StarMédica Hospital, Gastrointestinal and Liver Speciality Clinic, Mérida, Yucatán, México; 2 Universidad Autónoma de Yucatán, Facultad de Química, Biochemistry and Molecular Genetics Laboratory, Mérida, Yucatán, México; 3 Universidad Autónoma de Yucatán, Facultad de Química, Pharmacology Laboratory, México; 4 Universidad Autónoma de Yucatán, Facultad de Química, Drug Information Center, México

**Keywords:** lipids, liver enzymes, liver disease, steatosis, fibrosis, fibroza, steatoza, enzimi jetre, lipidi

## Abstract

**Background:**

Nonalcoholic fatty liver disease (NAFLD) is one of the most common causes of chronic liver disease and is associated with various co-morbidities. Transient elastography (FibroScan®) is a non-invasive method to detect NAFLD using the controlled attenuation parameter (CAP). We aimed to evaluate the association of the lipid panel and aminotransferases concentrations with the presence or absence of steatosis and fibrosis.

**Methods:**

One hundred and five patients with NAFLD were included. Hepatic steatosis was quantified by CAP (dB/m) and liver stiffness by Kilopascals (kPa), these values were then analyzed against patient lipid panel and serum concentrations of the liver enzymes aspartate aminotransferase (AST) and alanine aminotransferase (ALT). A correlation and multiple regression were used. Mann-Whitney U test was used as non-parametric analysis.

**Results:**

We observed an association between hepatic steatosis and total cholesterol (B = 0.021, p = 0.038, Exp (B) = 1.021, I.C = 1.001-1.041) as well as serum triglycerides (B = 0.017, p = 0.006, Exp (B) = 1.018 and I.C = 1.005-1.030). Similarly, we found an association between significant hepatic fibrosis and lower concentrations of total cholesterol (B = -0.019, p = 0.005, Exp (B) = 0.982 I.C = 0.969-0.995) and elevated AST (B = 0.042, p = 3.25 × 10^-4^, Exp (B) = 1.043 I.C = 1.019-1.068) independent of age, gender and BMI.

**Conclusions:**

Our results suggest that, total cholesterol and triglyceride concentrations positively correlate with hepatic steatosis while significant hepatic fibrosis is associated with lower total cholesterol and higher AST concentrations.

## Abstract (sr)

**Uvod:**

Oboljenje nealkoholna masna jetra (NAFLD) je jedan od najčešćih uzročnika hroničnog oboljenja koje je praćeno nizom različitih komorbiditeta. Kratkotrajna elastografija (FibroScan®) je neinvazivni metod za otkrivanje NAFLD primenom kontrolisanog prigušenog parametra (CAP). Namera je bila da se proceni veza između koncentracija lipidnog panela i aminotransferaza sa prisustvom ili odsustvom steatoze i fibroze.

**Metode:**

U ispitivanje je uključeno sto pedeset pacijenata sa NAFLD. Hepatična steatoza je merena pomoću CAP (dB/m) a krutost jetre pomoću kilopaskala (kPA), da bi zatim ove vrednosti bile analizirane prema vrednostima lipidnog panela i serumskih koncentracija jetrenih enzima aspartat aminotransferaze (AST) i alanin aminotransferaze (ALT). Za procenu su korišćena korelacija i multipla regresija. Mann-Whitney U test je korišćen kao ne-parametarska analiza.

**Rezultati:**

Posmatrana je veza između hepatične steatoze i ukupnog holesterola (B = 0,021, p = 0,038, Exp (B) = 1,021, I.C = 1,001-1, 041) kao i serumskih triglicerida (B = 0,017, p = 0,006, Exp (B) = 1,018 i I.C = 1,005-1,030). Nađena je slična veza između značajne hepatične fibroze i nižih koncentracija ukupnog holesterola (B = -0,019, p = 0,005, Exp(B) = 0,982, I.C = 0,969-0,995) i povišene AST (B = 0,042, P = 3.25 × 10-4, Exp (B) = 1,043, I.C = 1,019-1,068) nezavisno od starosti, pola i BMI.

**Zaključak:**

Dobijeni rezultati pokazuju da su koncentracije ukupnog holesterola i triglicerida u pozitivnoj korelaciji sa hepatičnom steatozom dok je značajna hepatična fibroza u vezi sa sniženim ukupnim holesterolom i višim koncentracijama AST.

## Introduction

Non-alcoholic fatty liver disease (NAFLD) is one of the most common causes of chronic liver disease worldwide with a global prevalence of 25%. In NAFLD, isolated steatosis can progress to advanced stages with non-alcoholic steatohepatitis (NASH) and fibrosis, increasing the risk of cirrhosis and hepatocellular carcinoma [1]. NAFLD frequently coexists with various diseases such as type 2 diabetes mellitus (>70% of patients), obesity (30-100% of patients) and cardiovascular diseases (20-92% of patients) [1] [2] [3].

The liver plays a key role in lipid metabolism. It functions in both the uptake and degradation of chylomicrons as well as in the synthesis of lipoproteins and in lipogenesis. In NAFLD an imbalance occurs between the uptake, secretion and synthesis of lipids in the liver which causes an excessive accumulation of macro and/or microvesicles rich in triglycerides in hepatocytes, reaching at least 5% of the total liver weight [4] [5].

Although biopsy is the gold standard for the evaluation of hepatic steatosis and fibrosis, it is an invasive procedure that, in a minority of cases, can lead to serious complications. Transient elastography (FibroScan®) is a recently developed, non-invasive, sensitive, and specific method that uses certain physical and ultrasonographic parameters to quantify steatosis and liver fibrosis [6] [7] [8] [9] [10] [11] [12] [13].

Several studies have demonstrated the usefulness of the lipid panel as an indirect marker of chronic liver disease [14] [15] [16] [17]. However, there is little evidence showing the association of serum lipid concentrations and aminotransferases with certain non-invasive quantitative parameters of hepatic steatosis and fibrosis, such as transient elastography (FibroScan®) with controlled attenuation parameter. The present study aims to evaluate the association of the lipid panel and aminotransferases with the values of FibroScan® of NAFLD.

## Material and Methods

### Patients

This was a cross-sectional study including 105 patients over 18 years of age with NAFLD of varying severity covering the whole spectrum of disease (Isolated steatosis and liver fibrosis with/without steatosis), who were treated at the Gastrointestinal and Liver Speciality Clinic at Merida, Yucatan, Mexico between January 2014 and May 2019. The demographic characteristics, anthropometric measures, and laboratory tests such as serum triglyceride concentration, total cholesterol, high-density lipoprotein cholesterol (HDL-C), low-density lipoprotein cholesterol (LDL-C), aspartate aminotransferase (AST) and alanine aminotransferase (ALT), were obtained with the patient's consent and they were measured according to the manufacturer's instructions with standardized methods. Exclusion criteria included significant alcohol intake (≥20 g/day for women and ≥40 g/day for men), clinical evidence of viral or autoimmune hepatitis, drug-induced fatty liver, or other metabolic liver diseases (such as haemochromatosis or Wilson's disease). Also, patients who were being treated with lipid lowering drugs or that declined participation in the study were excluded. This study was designed and conducted according to the principles of the Declaration of Helsinki and was approved by a Local Institutional Ethics Committee (MF-3002 Research Committee Hospital Hispano, Guadalajara, Mexico). Informed consent was obtained from each participant.

### Measurement of hepatic steatosis and fibrosis

The measurement of hepatic steatosis was performed through the CAP expressed in decibels per meter (dB/m) and the determination of fibrosis was performed through liver stiffness expressed in kPa, both measurements were made through Fibro Scan® transient elastography (FibroScan 501, Echo sens, Paris, France). The FibroScan® is a non-invasive method with high sensitivity and specificity for determining liver stiffness and steatosis and it has been previously described and validated in populations with NAFLD [6]. Measurements were performed by placing the tip of the transducer of the FibroScan® over the right lobe of the liver within intercostal spaces (typically between the ninth and eleventh intercostal space, at the mid-axillary line), with the patient lying in dorsal decubitus with the right arm in maximal abduction. This procedure was performed by expert personnel (>500 measurements) and reliability of the study was defined as ratio of the interquartile range (IQR)/median of liver stiffness measurement reflecting the variability of the total valid measures and should it not exceed 25%. Only examinations with at least 10 valid individual measurements were deemed valid. The presence of hepatic steatosis was defined by a CAP value ≥180 dB/m. Hepatic fibrosis was categorized as non-significant when liver stiffness was <7.9 kPa and significant when it was ≥7.9 kPa [7] [9] [12] [13].

### Statistical analysis

Data were reported as mean ± standard deviation for normal variables and median with interquartile range (25 and 75 percentile) for non-normal continuous quantitative variables. The distribution of the variables was analyzed using the Kolmogorov-Smirnov test. For statistical analysis in quantitative variables, the Student's t or Mann-Whitney U tests, according to data normality were used. The association between hepatic steatosis and liver fibrosis with lipid panel was assessed through univariate linear regression analysis. A multiple linear regression analysis was performed considering the variables with individual significance of the correlation analysis, where age, gender and body mass index (BMI) were considered covariates. The results were considered significant with a p value <0.05. The statistical program SPSS (IBM SPSS Statistics for Windows, version 25.0, Armonk, NY, USA) and GraphPad Prism (version 6.0; GraphPad Software, Inc., La Jolla, CA) for graphics, were used.

## Results

### Patient characteristics

A total of 105 patients were included, of which 59 (56%) were men. The mean age was 52.7±12.1 years old. Hepatic steatosis was present in 83 (79%) patients. According to liver stiffness, 35 (33.3%) patients showed significant hepatic fibrosis. The anthropometric, clinical and biochemical characteristics are shown in Table 1.

**Table 1:** Demographic, clinical, and laboratory parameters by gender The results are expressed as mean ± SD and median (25^th^ and 75^th^ percentile); BMI, body mass index; ALT, alanine aminotransferase; AST, aspartate aminotransferase; HDL-C, high-density lipoprotein cholesterol; LDL-C, low-density lipoprotein cholesterol.

Variable	All (n = 105)	Female (n = 46)	Male (n = 59)
Age (years)	52.7±12.1	59.9±11.9	49.0±10.9
BMI (kg/m^2^)	30.8±5.2	31.6±6.3	30.2±4.0
Fasting glucose (mmol/L)	5.5 (5.1–6.0)	5.5 (5.0–6.4)	5.6 (5.2–6.0)
ALT (U/L)	44.5 (32.3–64.5)	38.0 (25.5–60.5)	46.0 (35.0–66.0)
AST (U/L)	36.5 (26.3–54.8)	46.0 (28.0–58.4)	34.0 (26.0– 45.0)
Total cholesterol (mmol/L)	5.0±1.1	4.9 ± 0.98	5.1±1.1
HDL-C (mmol/L)	1.2 (1.1–1.3)	1.2 (1.2–1.3)	1.2 (0.9–1.3)
LDL-C (mmol/L)	3.2 (2.8–3.4)	3.2 (2.9–3.3)	3.1 (2.7–3.5)
Triglycerides (mmol/L)	1.7 (1.3–2.4)	1.6 (1.4–2.1)	2.0 (1.1–3.3)
Hepatic steatosis (dB/m)	287.8±57.8	279.5 ± 58.7	293.8±57.0
Liver stiffness (kPa)	7.1 (4.8 –14.3)	12.1 (5.3 –18.1)	6.1 (4.6–8.1)

**Figure 1:**
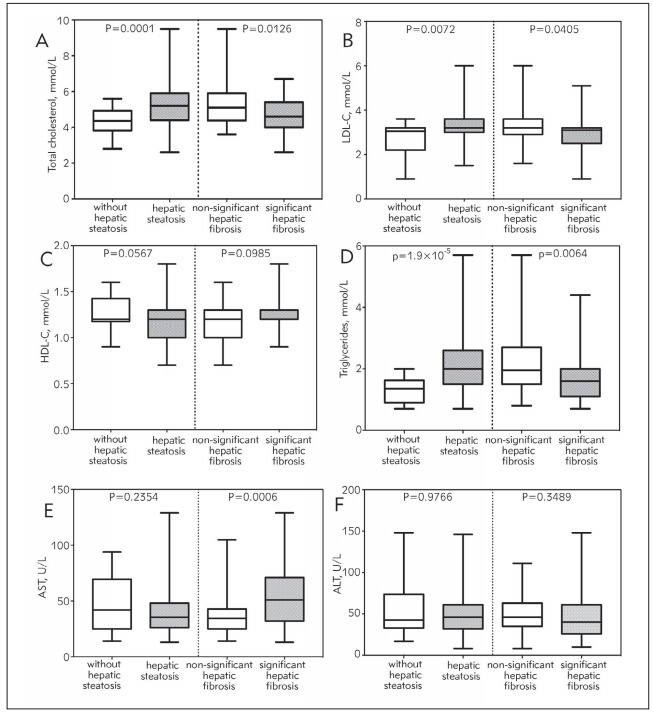
Boxplot graphic of median with interquartile range of (A-D) lipid panel and (E) aspartate aminotransferase (AST) and (F) alanine aminotransferase (ALT), according to hepatic steatosis and significant hepatic fibrosis The vertical line represents the division between the hepatic steatosis and advanced fibrosis analyses. P values were determined by the Mann-Whitney U test.

Total cholesterol (p=0.0001), LDL-C (p=0.0072) and triglycerides (p=1.9x10^-5^) serum concentrations were significantly higher in patients with hepatic steatosis. However, steatosis was not associated with significant differences in aminotransferase values. Regarding liver stiffness, patients with significant hepatic fibrosis showed significantly lesser serum concentrations of total cholesterol (p=0.0126), LDL-C (p=0.0405) and triglycerides (p=0.0064) than those with non-significant hepatic fibrosis. AST values (p=0.0006) were significantly higher in patients with significant hepatic fibrosis. Boxplot graphic of medians with interquartile range are shown in Figure 1.

Due to the correlation between hepatic steatosis and the concentrations of total cholesterol, LDL-C, and triglycerides a multiple linear regression analysis was carried out, observing an association with total cholesterol; B = 0.021, p = 0.038, Exp (B) = 1.021 and I.C = 1.001–1.041 and with triglycerides concentrations; B = 0.017, p = 0.006, Exp (B) = 1.018 and I.C = 1.005–1.030, independently of age, gender, and body mass index (BMI) (Table 2).

**Table 2:** Association of hepatic steatosis with total cholesterol and triglycerides B, regression coefficient; S.E, standard error, Sig, significance; Exp (B), odds ratio; C.I, confidence interval. Dependent variable, (hepatic steatosis); independent variables, (total cholesterol, triglycerides).

Variable	B	S.E	Sig.	Exp(B)	C.I 95%
Total Cholesterol	0.021	0.010	0.038	1.021	1.001–1.041
Triglycerides	0.017	0.006	0.006	1.018	1.005–1.030

Similarly, an association between significant hepatic fibrosis and concentrations of total cholesterol, LDL-C, triglycerides and AST was also evaluated. The results showed that the presence of significant hepatic fibrosis was associated with decreased total serum cholesterol; B = -0.019, p = 0.005, Exp (B) = 0.982 and I.C = 0.969–0.995; and elevated AST; B = 0.042, p = 3.25 x 10^-4^, Exp (B) = 1.043 and I.C = 1.019–1.068. These relationships were independent of age, gender, and BMI (Table 3).

**Table 3:** Association between significant hepatic fibrosis with total cholesterol and AST B, regression coefficient; S.E, standard error; Sig, significance; Exp(B), odds ratio; C.I, confidence interval. Dependent variable, (significant hepatic fibrosis); independent variables, (total cholesterol; AST, aspartate aminotransferase).

Variable	B	S.E	Sig.	Exp(B)	C.I 95% Exp(B)
Total Cholesterol	-0.019	0.007	0.005	0.982	0.969–0.995
AST	0.042	0.012	3.25 × 10^-4^	1.043	1.019–1.068

## Discussion

In clinical practice, the diagnosis of NAFLD is typically established through the combination of laboratory and radiographic findings with liver biopsy being the gold standard. However, these methods have limitations and provide a qualitative picture of steatosis and fibrosis of the liver that limit the reproducibility and practicality of the diagnosis [4]. Recently, non-invasive methods have been described for the quantification and surveillance of steatosis and stiffness such as the CAP and kPa of FibroScan®, which have demonstrated a strong correlation with the accumulation of fat and fibrosis in the liver [7] [10] [12] [13].

The results of our study showed that total cholesterol, triglycerides and serum LDL-C concentration were significantly higher in patients with hepatic steatosis. Observational studies such as DeFilippis et al. [18] and Loria et al. [19], showed that patients with NAFLD had a lipid profile consistent with an atherogenic phenotype showing dose-dependent characteristics such that the more severe their hypertriglyceridemia, the more severe their hepatic steatosis. The pathogenesis of NAFLD is one of insulin resistance, an imbalance in the metabolism of carbohydrates and lipids, and lipotoxicity that leads to hepatocellular damage, inflammation and fibrosis [14] [15]. The accumulation of fat in the form of lipid droplets in liver tissue is a fundamental histopathological characteristic for the diagnosis of NAFLD. Most commonly these lipid droplets are composed mainly of triglycerides [16] [17] [20]. Moreover, diet patterns also main role in NAFLD independently hyperlipidemia, diabetes and obesity. Musso et al. [21] found that subjects with NAFLD consumed a diet richer in saturated fatty acids and poorer in polyunsaturated fatty acids, fiber, and antioxidant vitamins C and E compared with controls. In addition, Yasutake et al. [22] [23] found that non-obese patients with NAFLD had significantly greater intake of dietary cholesterol and significantly lower intake of polyunsaturated fatty acids compared with an obese group with NAFLD. The main feature of dyslipidemia in patients with NAFLD is a normal LDL-C atherogenic lipid profile that consists of high levels of triglycerides, low levels of HDL-C and an increase in small and dense LDL particles. However, in this study there was a correlation observed between hepatic steatosis and LDL-C. Despite was a correlation observed between hepatic steatosis and LDL-C, this not was associated after a multiple linear regression analysis, perhaps LDL-C underestimates the true cholesterol load in NAFLD since their concentrations do not completely capture the total mass of lipoprotein particles [24].

On the other hand, we observed a significantly lower concentration of total serum cholesterol, triglycerides, and LDL-C in patients with significant hepatic fibrosis. After adjusting for age, gender and BMI, only total cholesterol and AST values remained significant. These findings can be explained by a decline in the liver's ability to capture, synthesize and mobilize lipoproteins and triglycerides due to the loss of hepatocytes in patients with significant hepatic fibrosis [25]. Similar results have been previously described by Tietge et al. [26] in a cohort of 52 patients with cirrhotic livers and 16 patients with clinically stable liver transplants. They found that patients with cirrhosis had a reduction in the clearance of free fatty acids and a diminished ability to synthesize cholesterol and triglycerides when compared to transplant patients, indicating the loss of primary liver function. In contrast to the lipid panel, AST and ALT are commonly used as indicators of hepatocellular injury [27]. The evidence has shown than elevations in AST confer an increased risk of all-cause mortality [28]. In NAFLD, AST elevations are frequent, but they are more pronounced in the presence of significant hepatic fibrosis and cirrhosis [27]. Similar results were found in our study.

Foremost among the limitations is that due to the transverse nature of cross-sectional studies like ours, the findings are correlational only in nature and do not establish causality.

In conclusion, our results suggest that total serum cholesterol and triglyceride concentrations are positively correlated with hepatic steatosis while significant hepatic fibrosis is associated with lower total cholesterol and higher AST concentrations in patients with NAFLD according to FibroScan® results. These findings suggest that the lipid panel and aminotransferases studies may serve as simple and useful metabolic markers for the identification and follow-up surveillance of those individuals at risk for NAFLD. Large prospective cohort studies are still needed in order to validate and confirm our results.

### Acknowledgements

To Jonathan Patrick Alessi for the critical analysis and the English revison. Eloiza Narváez for data organization and Hepatoescaner del sureste SCP for the facilitation of ethical access to FibroScan® data.

### Funding

The author received no financial support for the research.

### Ethical approval

MF-3002 (Research Committee Hospital Hispano, Guadalajara, Mexico).

### Contributor ship

C.C.L, I.C.M.E and L.R.J. conceived the presented idea and wrote the paper. M.G.I performed the analysis of steatosis and fibrosis.

T.R.J, A.A.V. and R.C.M. verified the analytical methods. All authors discussed the results and contributed to the final manuscript.

## Conflict of interest statement

The authors declare that they have no conflicts of interest in this work.

## List of abbreviations

NAFLD, Nonalcoholic fatty liver disease; CAP, Controlled attenuation parameter; kPa, Kilopascals; AST, Aspartate aminotransferase; ALT, Alanine aminotransferase; NASH, Non-alcoholic steatohepatitis; HDL-C, high-densitylipoprotein cholesterol; LDL-C, low-density lipoprotein cholesterol; IQR, interquartile range; BMI, Body mass index; B, regression coefficient; S.E, standard error; Sig, significance; Exp (B), Odds ratio; C.I, confidence interval.
